# Comparison between In-Hospital and Out-of-Hospital Acute Myocardial Infarctions: Results from the Regional Myocardial Infarction Registry of Saxony-Anhalt (RHESA) Study

**DOI:** 10.3390/jcm12196305

**Published:** 2023-09-29

**Authors:** Mohamad Assaf, Daniela Costa, Janka Massag, Christoph Weber, Rafael Mikolajczyk, Sara Lena Lückmann

**Affiliations:** Institute for Medical Epidemiology, Biometrics and Informatics, Interdisciplinary Center for Health Sciences, Medical School of the Martin-Luther University Halle-Wittenberg, Magdeburger Str. 8, 06112 Halle, Germany; mohamad.assaf@uk-halle.de (M.A.);

**Keywords:** myocardial infarction, in-hospital, out-of-hospital, complications, 30-day mortality, Germany

## Abstract

Aims: Risk factors and outcomes of in-hospital ST elevation myocardial infraction (STEMI) are well explored. Recent findings show that non-ST elevation myocardial infarction (NSTEMI) accounts for the majority of in-hospital infarctions (IHMIs). Our aim was to identify differences between IHMI and out-of-hospital myocardial infraction (OHMI) in terms of risk factors, treatment and outcomes, including both STEMI and NSTEMI. Methods: We analyzed the Regional Myocardial Infarction Registry of Saxony-Anhalt dataset. Patient characteristics, treatments and outcomes were compared between IHMI and OHMI. The association between clinical outcomes and myocardial infarction type was assessed using generalized additive models. Results: Overall, 11.4% of the included myocardial infractions were IHMI, and the majority were NSTEMI. Patients with IHMI were older and had more comorbidities than those with OHMI. Compared to OHMI, in-hospital myocardial infarction was associated with higher odds of 30-day mortality (OR = 1.85, 95% CI 1.32–2.59) and complications (OR = 2.36, 95 % CI 1.84–3.01). Conclusions: We provided insights on the full spectrum of IHMI, in both of its classifications. The proportion of IHMI was one ninth of all AMI cases treated in the hospital. Previously reported differences in the baseline characteristics and treatments, as well as worse clinical outcomes, in in-hospital STEMI compared to out-of-hospital STEMI persist even when including NSTEMI cases.

## 1. Introduction

Ischemic heart disease, specifically acute myocardial infraction (AMI), remains one of the leading causes of global mortality despite the decrease in prevalence and incidence [1]. Various medical and psychosocial risk factors for AMI have been identified, including coronary artery disease, heart failure, hyperlipidemia, obesity, smoking and substance abuse [2,3,4,5]. However, most of the previous studies included patients who developed AMI in the pre-hospital setting, otherwise known as out-of-hospital AMI (OHMI) [6,7,8]. Patients admitted to the hospital for cardiac or non-cardiac conditions may develop AMI during hospitalization, labeled as in-hospital AMI (IHMI), contributing considerably to the overall burden of the disease [6].

A number of studies have addressed this topic of IHMI but focused solely on STEMI. For example, a large US administrative analysis reported that in-hospital STEMI accounted for around 5% of total STEMIs, and it was associated with a 4-fold increase in in-hospital mortality [9]. Moreover, an Australian study confirmed that in-hospital STEMI was associated with higher 30-day mortality compared to out-of-hospital STEMI [10]. In Germany, a prospective study conducted in 1994–1997 in more than 50 hospitals revealed that 7% of all STEMI cases were in-hospital cases. The mortality of patients with an in-hospital STEMI was higher compared to patients who developed an out-of-hospital STEMI, at 27% vs. 14%, respectively [11]. Furthermore, resource utilization and in-hospital charges were found to be higher among patients with in-hospital STEMI compared to those with the out-of-hospital counterpart [9].

Recent investigations, however, showed that the majority of IHMIs tend to be non-ST-segment elevation MI (NSTEMIs) [6,12]. Thus, it is important to include NSTEMI when assessing the burden of IHMI, especially since the proportion of NSTEMIs is increasing over time [13]. Our aim was to identify differences between in-hospital and out-of-hospital AMIs, including both AMI classifications (STEMI and NSTEMI). We estimated the proportion of IHMIs among AMIs that are treated in hospitals in the state of Saxony-Anhalt, including both STEMIs and NSTEMIs. Additionally, we compared the baseline characteristics, treatments and outcomes between patients with IHMI and OHMI. Finally, we examined the association between the type of AMI (IHMI vs. OHMI) and clinical outcomes.

## 2. Methods

### 2.1. Study Design, Dataset Description and Data Collection

This is a cross-sectional study using data from The Regional Myocardial Infarction Registry of Saxony-Anhalt (German: Regionales Herzinfarktregister Sachsen-Anhalt, or RHESA). RHESA is a population-based registry including consecutively enrolled patients with AMI (2013–2019) from two regions in the federal state of Saxony-Anhalt, Germany. The study design has been described in detail elsewhere [14,15]. Due to the high mortality of AMI in this federal state (43% above the national average in 2012) [16] RHESA was founded in 2012 with the goal of identifying contributing risk factors and potential targets for intervention to improve health outcomes. It covers the rural region, Altmark, and the urban region, Halle (Saale). Individuals aged 25 years or older are included in the registry.

Three health departments, 16 hospitals, 16 residence registration offices, as well as centers for rescue services, participated in the registry. During hospitalization, trained physicians or study nurses reviewed the medical charts and collected, via a questionnaire, information related to sociodemographic factors, medical comorbidities, medical and procedural treatments, in-hospital complications and discharge status. The questionnaire was developed based on the Berlin Myocardial Infarction Registry questionnaire by Schuler et al. [17]. To track the survival status of AMI patients at 30 days, the study personnel contacted the participating registration offices at different points in time. In case of death, the cooperating health departments shared the death certificates with the RHESA registry. Our analysis, however, included only patients with AMI who arrived alive at a hospital for treatment, from 2013 to 2019. The exclusion criterion was missing information on the main variable of interest, “type of AMI” (N = 193). In Table S1 of the Supplementary Material, we present the characteristics and outcomes of the study population.

The average age at AMI occurrence was 70 years, and it occurred more frequently among males (62%). There was a high prevalence of classical and modifiable AMI risk factors in the study population (85% with hypertension, 51% with hyperlipidemia, 35% with diabetes, and 44% current or former smokers). Saxony-Anhalt is known to have a higher prevalence of cardiovascular risk factors compared to other German federal states [18]. As expected, the majority of the AMI cases in our sample were classified as NSTEMI rather than as STEMI (62% vs. 38%). Around 70% of all included patients who were treated with percutaneous coronary intervention (PCI), the 30-day mortality rate amounted to 8.7%.

### 2.2. Ethical Consideration

RHESA was approved by the Ethics Committee of the Medical Faculty of the Martin Luther University Halle-Wittenberg (Nr.: 2020-188) and by the State Data Protection and Privacy Commissioner of Saxony-Anhalt.

### 2.3. Variables and Outcomes

In this study, we used the “Third universal definition of myocardial infarction” of the European Society of Cardiology, which defined AMI as any rise and/or fall of high-sensitivity cardiac troponin by at least one unit above the 99th percentile of the upper reference, accompanied by ischemic signs and symptoms [19]. These include ischemic symptoms, new-onset changes in the ST-segment T wave or new left bundle branch block on the electrocardiogram (EKG), presence of pathological Q waves, new loss of myocardium or abnormality of wall motion identified via imaging, or coronary thrombus as evident on angiography or autopsy. Variables related to AMI type (IHMI or OHMI), AMI classification (STEMI or NSTEMI), shock upon presentation, treatment (aspirin, heparin, P2Y12 inhibitor, GPIIb/IIIa inhibitor, thrombolysis, PCI or bypass surgery) and in-hospital complications (a binary variable defined as having or not having any intubation, another shock, resuscitation, re-infarct, stroke, severe bleeding or need for re-intervention) were collected via questionnaires filled out by medical doctors or study nurses in the hospitals. The questionnaires also included information on the patients’ sociodemographic and risk factors, such as age at AMI occurrence (in years), sex, region of residence (Altmark/rural or Halle/urban), body mass index (BMI) that was categorized into four groups (<25, 25–29, 30–35, >35 kg/m^2^), smoking status and pre-existing medical conditions (diabetes, hypertension, hyperlipidemia, stroke, atrial fibrillation, history of previous MI, chronic kidney disease, peripheral vascular disease and heart failure).

The outcomes were 30-day mortality and occurrence of in-hospital complications.

### 2.4. Statistical Analysis

Patients were dichotomized based on AMI type (IHMI and OHMI). Categorical variables were reported in the form of frequencies (percentages) and 95% CI of the percentage. Numerical variables were reported in the form of mean (standard deviation) and 95% CI of the mean. Multiple imputation was applied for variables with missing values in more than five percent of all cases, which included body mass index, history of previous AMI, hyperlipidemia, hypertension, heart failure, peripheral vascular disease, chronic kidney disease and smoking status. Using a fully conditional specification method, forty complete datasets were generated based on the rule that the number of imputations should be equal to the percentage of missing cases or greater [20]. Since age could have non-linear associations with the outcomes, a generalized additive model (GAM) using the binomial family was applied to identify variables associated with AMI type (IHMI vs. OHMI) and outcomes (30-day mortality and in-hospital complications). Age showed a non-linear association with AMI type, which is depicted graphically in Figure S1 of the Supplementary Materials.

We identified two inflection points (55 years and 80 years). Therefore, we performed segmented logistic regression to calculate adjusted odds ratios (ORs) for the segments <55 years, 55–80 years and >80 years in model A (dependent variable was AMI type).

For the dependent variables, 30-day mortality and in-hospital complications occurrence, GAM analysis revealed a linear association with age (results not shown). Hence, no segmentation was needed for age in the logistic regression models B and C for the outcomes 30-day mortality and in-hospital complications, respectively. ORs and 95% confidence intervals (CIs) were reported. We adjusted for the following confounders: AMI type (in models B and C for 30-day mortality and in-hospital complications), age, male sex, BMI (reference: <25 kg/m^2^), region (reference: Altmark), AMI classification (reference: STEMI), smoking status, diabetes, hypertension, dyslipidemia, stroke, atrial fibrillation, history of previous AMI, chronic kidney disease, peripheral vascular disease and heart failure, based on previous studies. All statistical analyses were conducted using R-Studio version 4.2.1, Posit, PBC. Boston, MA, USA [21,22].

## 3. Results

The number of patients with AMI who were included in the study was 4272. Of those, 487 patients (11.4%) had IHMI, and the remaining had OHMI. Patients with IHMI were (on average) older than those with OHMI (72.28 ± 12.26 vs. 69.94 ± 13.36 years). There was no difference in the sex distribution between the two groups. Compared to patients with OHMI, patients with IHMI had a higher proportion of the following comorbidities: diabetes, hyperlipidemia, history of stroke, atrial fibrillation, heart failure, chronic kidney disease and peripheral vascular disease (Table 1). The proportion of patients with IHMI among all patients with AMI in the rural region (Altmark) was lower than in the urban region (Halle), at 9.2%, 95% CI: 8.0–10.5 vs. 13.6%, 95% CI: 12.2–15.0, respectively (row percentages, not shown in the table).

NSTEMI was more common in patients with IHMI compared to patients with OHMI (75.4%, 95% CI: 71.4–79.0 vs. 60.3%, 95% CI: 58.8–61.9). Of the total 2650 NSTEMI cases, 367 (13.8%) were in-hospital. Of all the included STEMI cases, 7.4% were in-hospital. In terms of treatment, patients with IHMI received treatment with anti-platelets (aspirin or P2Y12 inhibitor) or heparin less frequently. Nearly 2% of the patients in each group received thrombolysis treatment. A considerably smaller proportion of patients with IHMI were managed with PCI (56.7%, 95% CI: 52.2–61.0 vs. 71.1%, 95% CI: 68.7–71.6 in the OHMI group). The IHMI group had a higher proportion of in-hospital complications (14.6%, 95% CI: 13.5–15.8 vs. 29%, 95% CI: 25.1–33.1]. Considering the main outcome, patients with IHMI had a higher 30-day morality rate than patients with OHMI (12.5%, 95% CI: 9.8–15.7 vs. 8.2%, 95% CI: 7.4–9.2) (Table 2).

Patients with IHMI were 1.92 times more likely to have NSTEMI than STEMI (95% CI: 1.52–2.46) compared to patients with OHMI. In addition, patients with IHMI were more likely to have heart failure, chronic kidney disease and peripheral vascular disease compared to patients with OHMI. Residents of the urban region (Halle) were 1.44 times more likely to experience IHMI than OHMI compared to residents of the rural region (Altmark) (95% CI: 1.14–1.83) (Table 3), consistent with the crude difference in proportions.

For each of the two dependent outcomes, 30-day mortality and in-hospital complications, we adjusted for medical comorbidities and other confounders in the multivariable analysis. We found that the adjusted odds of 30-day mortality was higher among patients with IHMI compared to patients with OHMI (OR = 1.81, 95% CI: 1.29–2.54) (Table 4). IHMI was also associated with a higher adjusted odds of in-hospital complications (OR = 2.35, 95% CI: 1.84–3.01) (Table 5).

## 4. Discussion

The aim of this study was to identify differences between IHMI and OHMI in terms of risks factors and health outcomes, including both classifications (STEMI and NSTEMI), while avoiding the limitations of previous investigations. For this purpose, we estimated the proportion of IHMI among STEMI and NSTEMI cases managed in the hospitals in the regions of this study, and compared the characteristics, treatments and clinical outcomes between patients with IHMI and those with OHMI.

We found that 11.4% of the AMI cases in our sample were IHMI, with the majority being classified as NSTEMI. Patients with IHMI were older and had more comorbidities than those with OHMI. Additionally, they were less frequently managed with PCI. Patients with IHMI had higher 30-day mortality and higher proportion of complications, which did not change after adjusting for relevant confounders. Previous studies had been conducted to identify the potential cause of IHMI. The majority of IHMI cases among patients hospitalized for non-cardiac conditions were attributed to perioperative AMI [23], contributing to 50% of the cases. The PeriOperative ISchemic Evaluation trial (POISE) involving 23 countries reported a perioperative AMI incidence of 5% within 30 days of the random assignment date to the control or intervention group [24]. Furthermore, some cardiac [25,26], infectious [27,28] and metabolic diseases [29,30], as well iatrogenic causes [31,32,33], can lead to AMI as an in-hospital complication.

When considering the STEMI cases only, our estimate of in-hospital STEMI (7.4%) was found to be comparable to the results of previous investigations. Similar to our results, Zahn et al. demonstrated that 7% of STEMI cases treated in hospitals in Germany between 1994 and 1997 were IHMI [11], and Kaul et al. reported that 5% of the STEMI cases included in the California State Inpatient Database were in-hospital [9]. Both of these studies did not include NSTEMI. This is important since IHMI cases often tend to be NSTEMI, as evident in our study (75%) and supported by previous studies. Maynard et al. reported that 9.5% of the IHMI cases in their analysis were initially diagnosed as STEMI, and the remaining 90.5% were diagnosed as NSTEMI [12]. Additionally, Bradley et al. found that 85.4% of the identified IHMI cases in US Veterans Health Administration facilities were classified as NSTEMI [6]. Nonetheless, these proportions should be regarded with caution. Bradley et al. examined the incidence of IHMI in a case–control study, with the control group comprising any hospital admission with a diagnosis different from ischemic heart disease, rather than an OHMI group [6]. Maynard et al. examined the differences between IHMI and OHMI including both STEMI and NSTEMI, with a reported IHMI prevalence of 11.2%, which was similar to our result (11.4%) [12]. However, post-operative IHMI cases, which could contribute to 5% of total IHMI cases, were excluded [12,24,31]. A Swiss study also including both STEMI and NSTEMI used nationwide registry data and identified a substantially lower proportion of IHMI, amounting to 1% only, possibly due to lack of systematic inclusion of this AMI type in the study registry [31]. On the other hand, our analysis included, per design, only those patients who arrived at the hospital alive in the OHMI group. Patients who were found dead and later classified as AMI or who could not be successfully revived and died after their arrival to the emergency department were excluded from the analysis. With respect to the total number of AMI cases, IHMI fraction would be in fact lower.

Comparing the characteristics between patients with IHMI and OHMI, we found that patients with IHMI constituted an older and more comorbid subgroup of patients with AMI. Heart failure, chronic kidney disease and peripheral vascular disease were associated with a higher risk of IHMI, after adjusting for various confounders. This is consistent with the risk factors that were found to be associated with a higher risk of in-hospital STEMI in the multivariable analysis by Kaul et al. [9]. Patients with in-hospital STEMI were 1.7 times more likely to have preexisting heart failure (95% CI: 1.5–1.9), 1.2 times more likely to have kidney failure (95% CI: 1.04–1.3), and 1.5 times more likely to have peripheral vascular disease (95% CI: 1.3–1.6), relative to patients with out-of-hospital STEMI.

Previous studies that included both STEMI and NSTEMI did not perform a multivariable analysis to identify independent risk factors of IHMI. However, they confirmed that older age and comorbidities (such as diabetes, hyperlipidemia, history of stroke, atrial fibrillation, heart failure, chronic kidney disease and peripheral vascular disease) were more common in patients with IHMI, compared to those with OHMI [12,31]. Interestingly, Erne at al. reported that IHMI was more frequent among females, in contrast to our study where we found no difference between the two sexes [31]. We believe that this lowers the risk of bias in our study, given the established differences in the prevalence of AMI risk factors and outcomes between men and women [34,35].

In terms of clinical outcomes, studies including both STEMI and NSTEMI showed that in-hospital complications (in particular, bleeding, cardiogenic shock and cardiac arrest) were more frequent among patients with IHMI compared to those with OHMI [12]. Nevertheless, no adjustment for confounders was performed. We demonstrated that patients with IHMI had a higher adjusted odds of in-hospital complications compared to OHMI patients, even after adjusting for chronic medical comorbidities. The association between IHMI and a higher risk of in-hospital complications was reported in studies including STEMI only. Patients with in-hospital STEMI were at a higher risk of developing shock, major bleeding, stroke and major adverse cardiac events, relative to those with out-of-hospital STEMI [9,23,36]. Considering mortality associated with IHMI, we found that the 30-day mortality rate in patients with IHMI was higher compared to patients with OHMI, even after adjusting for age, comorbidities and EKG classification. This is consistent with the findings of previous studies on the association between IHMI and short-term AMI mortality, while including both STEMI and NSTEMI. For example, Erne et al. reported a higher adjusted odds of in-hospital mortality among patients with IHM compared to patients with OHMI (OR = 2.4, 95% CI: 1.6–3.4) [31]. Additionally, Maynard et al. found that compared to OHMI, IHMI was associated with a higher in-hospital mortality rate (27% vs. 9%), as well as a higher odds for 30-day mortality after adjusting for medical comorbidities and EKG diagnosis (OR = 2.0, 95% CI: 1.7–2.4) [12].

Four potential explanations have been suggested to justify the worse clinical outcomes among patients with IHMI compared to those with OHMI. First, older age and higher prevalence of pre-existing medical conditions among patients with IHMI are associated with atypical AMI presentations, leading to delays in diagnosis and subsequent treatment [23,36,37]. Second, the worse clinical condition and severity of the primary disease (underlying reason for hospitalization) in patients with IHMI can contribute to higher risks of morbidity and mortality in this group. In our study, we accounted for various confounding comorbidities, but it was possible residual confounding factors remained. Third, patients with IHMI received PCI less frequently than patients with OHMI, potentially contributing to their higher mortality [9]. Unfortunately, information related to coronary angiography and its findings was not available. This could have an impact on the choice of treatment strategy and clinical outcomes. Previous studies including STEMI cases only reported that patients with in-hospital STEMI undergo coronary angiography less commonly comparted to patients with out-of-hospital STEMI [23,37,38]. In addition, a considerably lower proportion of in-hospital STEMI cases (less than 56%) are managed with PCI in comparison to out-of-hospital cases. Despite adjusting for comorbidities and other confounders, patients with in-hospital STEMI were found to have a lower odds of undergoing coronary angiography and PCI [9]. However, when considering only patients who underwent coronary angiography, Jaski et al. found no difference in the proportion of performed intervention between in-hospital and out-of-hospital STEMI cases [38]. As for previous investigations that included both STEMI and NSTEMI, lower proportions of PCI were also reported in patients with IHMI compared to patients with OHMI [12,31]. The most common reason for not performing coronary angiography and subsequent PCI is high bleeding risk, followed by other factors like neurological and cognitive dysfunction, patients’ preferences and severe comorbidities (such as pulmonary embolism or bowel ischemia) [23,38]. Another reason is that some IHMI cases result from pathophysiological mechanisms not involving atherosclerotic rupture and thrombus formation, known as type 2 AMI. This may be indicated by the presence of common risk factors between IHMI and type 2 AMI, such as perioperative stress, heart failure arrhythmia and sepsis. In such cases of IHMI, PCI is less often performed [6,39]. Interestingly, a study by Jaski et al. showed differences in factors influencing the ineligibility for PCI between patients with in-hospital and out-of-hospital STEMI [38]. While the risk of bleeding was the most common reason for PCI ineligibility in the in-hospital STEMI group, the most common reason in the out-of-hospital STEMI group was complex coronary artery disease. Regarding NSTEMI, there are currently no available studies comparing the patterns of PCI utilization and differences in ineligibility between in-hospital NSTEMI and its out-of-hospital counterpart.

Finally, in some cases such as perioperative IHMI, patients are admitted to clinical wards other than internal medicine. Medical staff in other wards may be less trained in early recognition of AMI and initiation of the necessary work-up, especially since IHMI is relatively uncommon and its clinical presentation may be atypical, as previously mentioned.

This study has some limitations. Despite substantial effort, no complete coverage of AMIs in the considered regions was achieved. Furthermore, the two regions of Saxony-Anhalt might not provide representative estimates for the federal state. About 33% of the population lives in rural regions in Saxony-Anhalt [40], while in the registry, the rural and urban regions contribute equally. Additionally, there might be some variation among the rural regions in Saxony-Anhalt regarding mortality and likely morbidity of AMI. Another limitation is that our analysis is based on routine clinical data; thus, some inherent problems of data quality and availability, as well as limited standardization, could be present. This includes information related to the reason for hospitalization is not available. Finally, we could not adjust for the different kinds of coronary lesions identified during coronary angiography, which could influence the outcomes.

Nonetheless, to our knowledge, this is the first study conducted in Germany to assess IHMI, including NSTEMI and STEMI. The substantial sample size and the inclusion of urban and rural regions constitute additional strengths.

## 5. Conclusions

We estimated the proportion of IHMI to be at around one ninth of all AMI cases treated in hospitals based on the data of the regional AMI registry of Saxony-Anhalt, with a 75% chance of being classified as NSTEMI. Previously reported differences in baseline characteristics and treatments, as well as worse clinical outcomes, in in-hospital STEMI compared to out-of-hospital STEMI persist even when including NSTEMI cases. Patients with IHMI in our study tended to be older and had more comorbidities than patients with OHMI. Despite adjustment for these differences, IHMI was associated with a higher risk of in-hospital complications and mortality. This can be a consequence of differences in clinical condition beyond the available information (residual confounding) or difficulties in early diagnosis and proper management of IHMI. In conclusion, our study provides insights on the full spectrum of in-hospital AMI, in both of its classifications, and shows that NSTEMI is the bigger contributor to this medical entity and, thus, may play a bigger role in the clinical course of in-hospital AMI.

## Figures and Tables

**Table 1:** Distribution of patients’ sociodemographic parameters and risk factors based on AMI type.

	OHMI		IHMI	
	N (%) or Mean (SD)	95% CI	N (%) or Mean (SD)	95% CI
Total = 4272	3785 (88.6)		487 (11.4)	
Sociodemographic factors				
Age (years)	69.39 (13.36)	68.97–69.82	72.23 (12.29)	71.1–73.3
Male	2475 (65.4)	63.9–66.9	310 (63.7)	59.3–67.8
Altmark (rural)	1903 (50.3)	48.7–51.9	192 (39.4)	35.2–43.8
Halle (urban)	1882 (49.7)	48.1–51.3	295 (60.6)	56.2–64.8
Risk factors				
Body mass index (kg/m^2^)				
<25	772 (20.4)	19.1–21.7	88 (18.1)	14.8–21.8
25–<30	1911 (50.5)	48.9–52.1	246 (50.5)	45.9–55.0
30–35	864 (22.8)	21.5–24.2	118 (24.2)	20.5–28.3
>35	238 (6.3)	5.5–7.12	35 (7.2)	5.1–9.9
Previous AMI	614 (16.2)	15.1–17.4	86 (17.7)	14.5–21.2
Diabetes	1289 (33.6)	32.6–35.6	202 (41.1)	37.2–45.9
Hypertension	3237 (85.5)	84.4–86.6	411 (84.4)	81.0–87.4
Hyperlipidemia	1976 (52.2)	50.6–53.8	209 (42.9)	38.6–47.3
Stroke	343 (9.4)	8.2–10.0	63 (13.6)	10.2–16.1
Atrial fibrillation	655 (17.9)	16.1–18.5	139 (29.8)	24.7–32.7
Heart failure	795 (21)	19.7–22.3	166 (34.1)	30.0–38.4
Chronic kidney disease	938 (24.8)	23.4–26.2	201 (41.3)	37.0–45.7
Peripheral vascular disease	374 (9.9)	9.0–10.9	101 (20.7)	17.3–24.5
Non-smokers	2117 (55.9)	54.3–57.5	271 (55.6)	51.2–60.0
Smokers	1175 (31)	29.6–32.5	142 (29.2)	25.3–33.3
Former smokers	493 (13)	12.0–14.1	74 (15.2)	12.2–18.6

Numerical variables are presented in the form of mean (standard deviation) and 95% CI of the mean. Categorical variables are shown in the form of frequency (%) and 95% CI of the percentage. SD: standard deviation. CI: confidence interval. OHMI: out-of-hospital myocardial infarction. IHMI: in-hospital myocardial infarction.

**Table 2:** Treatment and clinical outcomes of patients with AMI in Saxony-Anhalt based on AMI type.

	OHMI		IHMI	
	N (%) or Mean (SD)	95% CI	N (%) or Mean (SD)	95% CI
Total = 4272	3785 (88.6)		487 (11.4)	
Aspirin	3158 (83.4)	82.2–84.6	331 (68.0)	63.7–72.0
P2Y12 inhibitor	1548 (40.9)	39.3–42.5	167 (34.5)	30.4–38.8
GPIIb/IIIa inhibitor	26 (0.7)	0.5–1.0	3 (0.6)	0.2–1.06
Heparin	3004 (79.4)	78.1–80.6	321 (65.9)	61.6–70.0
Thrombolysis	73 (1.9)	1.5–2.4	9 (1.8)	0.9–3.3
PCI	2655 (70.1)	68.7–71.6	276 (56.7)	52.2–61.0
Bypass surgery	201 (5.3)	4.6–6.1	36 (7.4)	5.3–10.0
NSTEMI	2283 (60.3)	58.8–61.9	367 (75.4)	71.4–79.0
STEMI	1502 (39.7)	38.1–41.2	120 (24.6)	21–28.6
Complications	554 (14.6)	13.5–15.8	141 (29.0)	25.1–33.1
30-day mortality	312 (8.2)	7.4–9.2	61 (12.5)	9.8–15.7

Numerical variables are presented in the form of mean (standard deviation) and 95% CI of the mean. Categorical variables are shown in the form of frequency (%) and 95% CI of the percentage. SD: standard deviation. CI: confidence interval. PCI: percutaneous coronary intervention. NSTEMI: non-ST segment elevation myocardial infarction. STEMI: ST-segment elevation myocardial infarction.

**Table 3:** Factors associated with the odds of IHMI (reference: OHMI): model A.

Factors	Adjusted OR	95% CI
Age (in years)		
<55	0.99	0.96–1.02
55–80	1.035	0.99–1.08
>80	0.90	0.84–1.097
Male	0.93	0.74–1.16
Body mass index		
(reference group: <25 kg/m^2^)		
25–<30 kg/m^2^	1.27	0.95–1.69
30–35 kg/m^2^	1.35	0.97–1.87
>35 kg/m^2^	1.34	0.85–2.12
Halle (Altmark as reference)	1.44	1.14–1.83
NSTEMI (reference: STEMI)	1.92	1.52–2.46
Non-smokers	1	
Smokers	1.22	0.92–1.63
Previous smokers	1.12	0.81–1.54
Diabetes	1.11	0.89–1.39
Hypertension	0.86	0.64–1.18
Dyslipidemia	0.74	0.59–0.94
Stroke	1.06	0.76–1.45
Atrial fibrillation	1.24	0.96–1.60
History of previous AMI	1.11	0.89–1.39
Chronic kidney disease	1.86	1.6–2.4
Peripheral vascular disease	1.74	1.59–1.94
Heart failure	1.16	1.06–1.45

Variables included in the model: age, male sex, BMI (reference: <25 kg/m^2^), region (reference: Altmark), AMI classification (reference: STEMI), smoking status, diabetes, hypertension, dyslipidemia, stroke, atrial fibrillation, history of previous AMI, chronic kidney disease, peripheral vascular disease and heart failure. OR: odds ratio. CI: confidence interval. IHMI: in-hospital myocardial infarction. OHMI: out-of-hospital myocardial infarction. STEMI: ST-segment elevation myocardial infarction. NSTEMI: non-ST-segment elevation myocardial infarction. AMI: myocardial infarction.

**Table 4:** Factors associated with odds of 30-day mortality: model B.

Factors	Adjusted OR	95% CI
IHMI (reference: OHMI)	1.81	1.29–2.54
Age (in years)	1.06	1.05–1.08
Male	1.11	0.84–1.45
Body mass index		
(Reference group: <25 kg/m^2^)		
25–<30 kg/m^2^	1.17	0.84–1.63
30–35 kg/m^2^	1.07	0.72–1.59
>35 kg/m^2^	0.77	0.39–1.51
Halle (Altmark as reference)	1.05	0.80–1.40
STEMI (reference: NSTEMI)	2.39	1.84–3.12
Non-smokers		
Smokers	1.40	0.99–1.97
Previous smokers	0.68	0.43–1.10
Diabetes	1.35	1.04–1.75
Hypertension	1.01	0.67–1.52
Dyslipidemia	1.11	0.84–1.48
Stroke	1.63	1.16–2.39
Atrial fibrillation	1.17	0.87–1.58
History of previous AMI	0.79	0.55–1.13
Chronic kidney disease	1.40	1.04–1.88
Peripheral vascular disease	1.38	0.96–1.98
Heart failure	1.20	0.89–1.62

Variables included in the model: AMI type (reference: OHMI), age, male sex, BMI (reference: <25 kg/m^2^), region (reference: Altmark), AMI classification (reference: STEMI), smoking status, diabetes, hypertension, dyslipidemia, stroke, atrial fibrillation, history of previous AMI, chronic kidney disease, peripheral vascular disease and heart failure. OR: odds ratio. CI: confidence interval. IHMI: in-hospital myocardial infarction. OHMI: out-of-hospital myocardial infarction. STEMI: ST-segment elevation myocardial infarction. NSTEMI: non-ST-segment elevation myocardial infarction. AMI: myocardial infarction.

**Table 5:** Factors associated with odds of in-hospital complications: model C.

Factors	Adjusted OR	95% CI
IHMI (reference: out of hospital AMI)	2.35	1.84–3.01
Age (in years)	1.02	1.01–1.03
Male	0.90	0.74–1.10
Body mass index (kg/m^2^) (Reference: <25)		
25–<30	0.92	0.73–1.17
30–35	0.99	0.75–1.31
>35	1.07	0.71–1.61
Halle (Altmark as reference)	1.67	1.35–2.06
STEMI (reference: NSTEMI)	2.35	1.84–3.01
Non-smokers		
Smokers	1.02	1.01–1.03
Previous smokers	0.99	0.86–1.07
Diabetes	0.90	0.74–1.10
Hypertension	1.67	1.35–2.06
Dyslipidemia	2.35	1.84–3.01
Stroke	1.02	1.01–1.03
Atrial fibrillation	0.90	0.74–1.10
History of previous AMI	1.67	1.35–2.06
Chronic kidney disease	2.35	1.84–3.01
Peripheral vascular disease	1.02	1.01–1.03
Heart failure	0.90	0.74–1.10

Variables included in the model: AMI type (reference: OHMI), age, male sex, BMI (reference: <25 kg/m^2^), region (reference: Altmark), AMI classification (reference: STEMI), smoking status, diabetes, hypertension, dyslipidemia, stroke, atrial fibrillation, history of previous AMI, chronic kidney disease, peripheral vascular disease and heart failure. OR: odds ratio. CI: confidence interval. IHMI: in-hospital myocardial infarction. OHMI: out-of-hospital myocardial infarction. STEMI: ST-segment elevation myocardial infarction. NSTEMI: non-ST-segment elevation myocardial infarction. AMI: myocardial infarction.

## Data Availability

The datasets used and/or analyzed during the current study are available from the corresponding author upon request.

## Supplementary Materials

The following supporting information can be downloaded at: https://www.mdpi.com/article/10.3390/jcm12196305/s1, Table S1: Characteristics and outcomes of the study population. Figure S1: Association between age and AMI type (IHMI vs. OHMI).

## Abbreviations

AMI	acute myocardial infarction
BMI	body mass index
CI	confidence interval
IHMI	in-hospital myocardial infarction
NSTEMI	non-ST segment elevation myocardial infarction
SD	standard deviation
STEMI	ST-segment elevation myocardial infarction
OHMI	out-of-hospital myocardial infarction
OR	odds ratio
