# Supplementary material for: Comparison between In-Hospital and Out-of-Hospital Acute Myocardial Infarctions: Results from the Regional Myocardial Infarction Registry of Saxony-Anhalt (RHESA) Study
Source: J Clin Med. 2023 Sep 29;12(19):6305. doi: 10.3390/jcm12196305 (PMC10573894; doi:10.3390/jcm12196305)
Supplement: Supplementary file 1 [file jcm-12-06305-s001.zip › jcm-2622325-supplementary.pdf]

Table S1. of the Supplementary material S1: Characteristics and outcomes of the study population.

Total = 4272	Frequency (%) or mean (SD)
Age (years)	69.72 (13.27)
Male	2785 (62.5)
Altmark (Rural)<br><br>Halle (Urban)	2095 (49.0)<br><br>2177 (51.0)
Body mass index (kg/m^2)<br><25<br>25 - <30<br>30-35<br>>35	 <br>860 (20.1)<br>2157 (50.5)<br>982 (23.0)<br>273 (6.4)
Previous myocardial infarction	700 (16.4)
Diabetes	1486 (34.8)
Hypertension	3648 (85.4)
Hyperlipidemia	2185 (51.1)
Stroke	404 (9.5)
Atrial fibrillation	793 (18.6)
Heart failure	961 (22.5)
Chronic kidney disease	1139 (26.7)
Peripheral vascular	475 (11.1)
None smoker<br>Smoker<br>Former smoker	2388 (55.9)<br>1317 (30.8)<br>567 (13.3)
NSTEMI<br>STEMI	2650 (62.0)<br>1622 (38.0)
PCI	2931 (68.6)
Bypass surgery	237 (5.5)
Complications	695 (13.6)
30-day mortality	373 (8.7)

Numerical variables presented in the form mean (standard deviation), and categorical variables in the form frequency (%). SD: standard deviation. STEMI: ST-segment elevation myocardial infarction. NSTEMI: Non-ST-segment myocardial infarction. PCI: Percutaneous coronary intervention.

Figure S1. of the Supplementary material S1: Association between age and AMI type (IHMI vs OHMI)

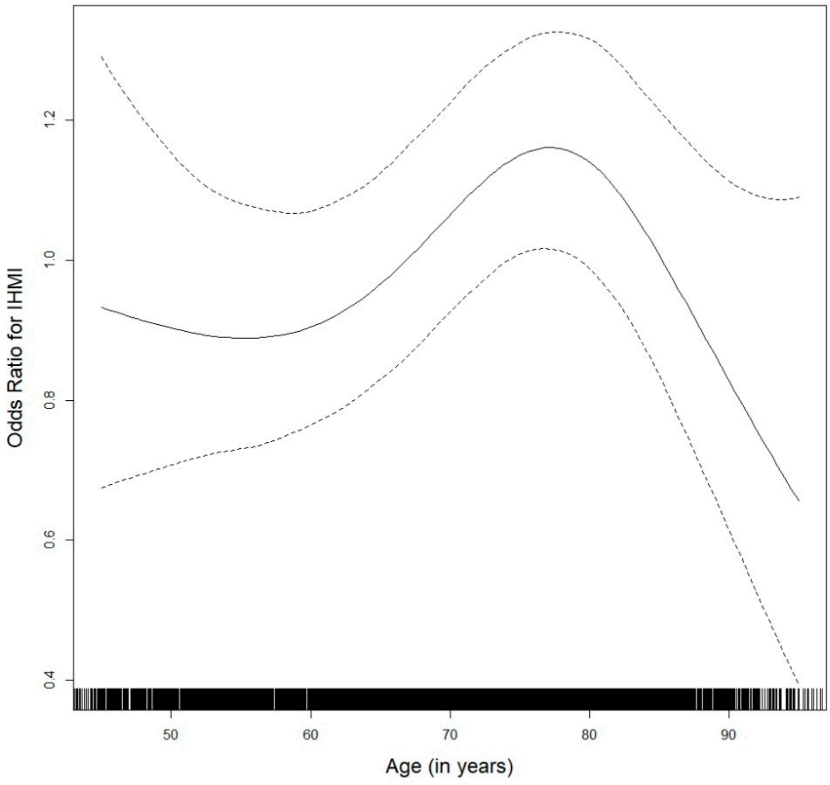

Figure S1 shows a non-linear association between age and AMI type, after adjusting for other confounders. Two inflection points can be identified: 55 years and 80 years. Therefore, segemented logistic regression was performed to calculate adjsuted odds ratios for the segments: < 55 years, 55-80 years and >80 years. Note: Full line = odds ratio, dotted line = 95% CI.
